# Uppermost crustal structure regulates the flow of the Greenland Ice Sheet

**DOI:** 10.1038/s41467-021-27537-5

**Published:** 2021-12-15

**Authors:** G. A. Jones, A. M. G. Ferreira, B. Kulessa, M. Schimmel, A. Berbellini, A. Morelli

**Affiliations:** 1 Department of Geography, Swansea University, Singleton Park, Swansea, UK; 2 Department of Earth Sciences, University College London, London, UK; 3 CERIS, Instituto Superior Técnico, Universidade de Lisboa, Lisboa, Portugal; 4 University of Tasmania, Hobart, TAS Australia; 5 Geosciences Barcelona (GEO3BCN-CSIC), Barcelona, Spain; 6 Istituto Nazionale di Geofisica e Vulcanologia, Sezione di Bologna, Bologna, Italy

**Keywords:** Climate change, Cryospheric science, Geology, Geophysics, Seismology

## Abstract

The flow of the Greenland Ice Sheet is controlled by subglacial processes and conditions that depend on the geological provenance and temperature of the crust beneath it, neither of which are adequately known. Here we present a seismic velocity model of the uppermost 5 km of the Greenlandic crust. We show that slow velocities in the upper crust tend to be associated with major outlet glaciers along the ice-sheet margin, and elevated geothermal heat flux along the Iceland hotspot track inland. Outlet glaciers particularly susceptible to basal slip over deformable subglacial sediments include Jakobshavn, Helheim and Kangerdlussuaq, while geothermal warming and softening of basal ice may affect the onset of faster ice flow at Petermann Glacier and the Northeast Greenland Ice Stream. Interactions with the solid earth therefore control the past, present and future dynamics of the Greenland Ice Sheet and must be adequately explored and implemented in ice sheet models.

## Abstract (web summary)

Anomalously slow seismic velocities in the upper Greenlandic crust reveal soft sedimentary substrates beneath major outlet glaciers. This, together with elevated geothermal heat flux observed at the onset of fast ice flow, has major implications for ice-sheet dynamics.

## Introduction

The Greenland Ice Sheet (GrIS) is the second largest reservoir of freshwater on Earth. Accelerated ice mass loss of the GrIS as a result of climatic forcing from the early 1990’s accounts for ~10% of mean global sea level rise^1^. Ice mass loss has increased over six fold from 34 Gt per yr in the period 1991–2001 to 215 Gt per yr in 2002–2011 and shows no sign of slowing down^1^. The basal geological conditions beneath an ice sheet or glacier are a fundamental control on ice flow with the substrate and the presence of liquid water being a key prerequisite for fast ice flow.

**Fig. 1:**
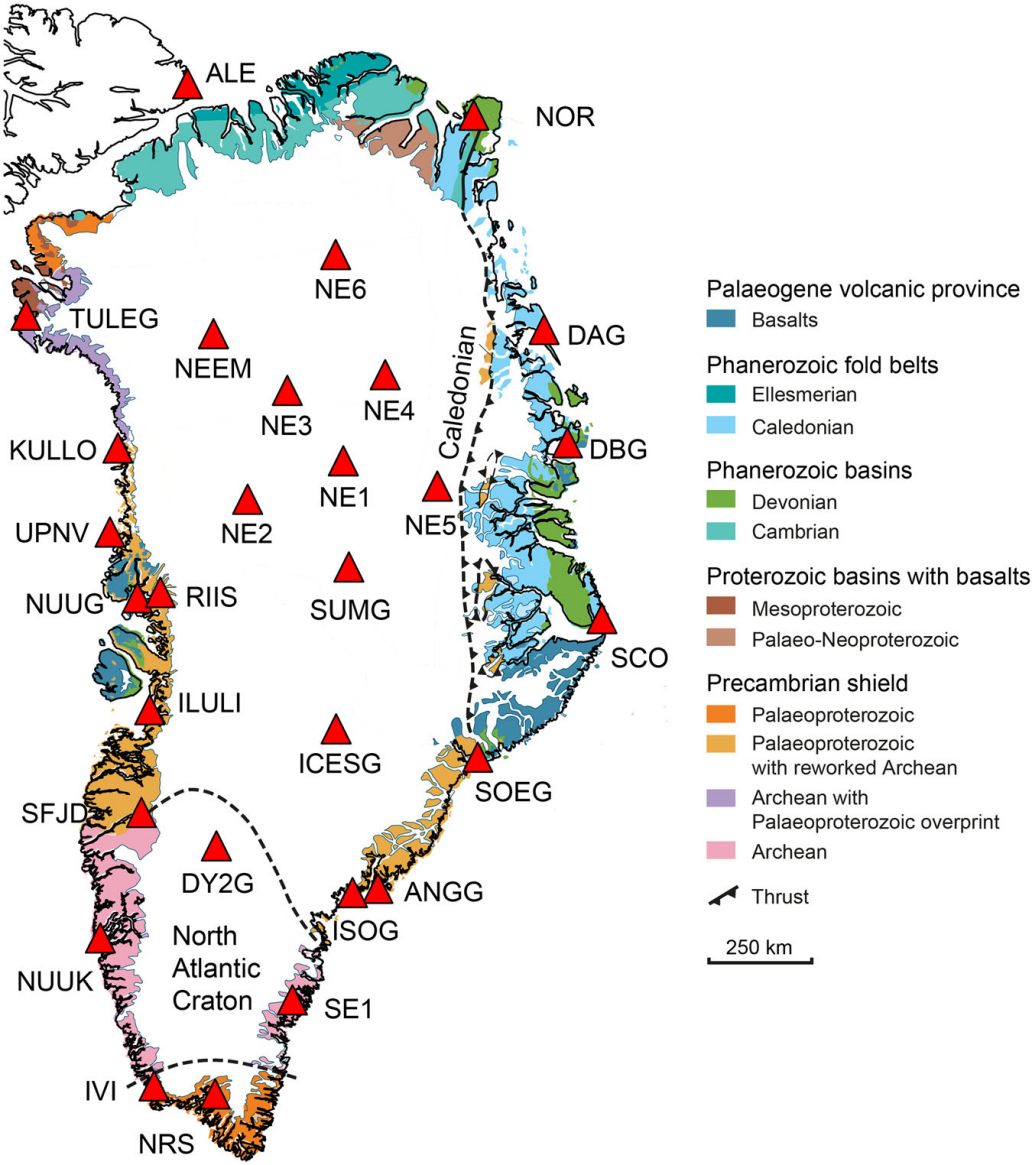
Geological map of Greenland. Geological units are shown in colour  (modified after Dawes^76^) and the red triangles are the locations of the seismic stations used in this study.

The Greenlandic crust primarily comprises an Archaean and early Proterozoic crystalline basement (Fig. 1), which was formed during a series of orogenic events that later stabilised to form a key component of the Laurentian shield^2^. Subsequent geological evolution has been restricted to the shield’s margins with the formation of extensive sedimentary basins to the north and north-east dating from the Precambrian to early Devonian. The sedimentary basins to the north and east were affected by late Palaeozoic orogenic events culminating in the east-west trending Ellesmerian Fold Belts to the north and the extensive north-south trending Caledonian-Appalachian orogenic belt along the east of Greenland^2^. Successive rifting events during the late Devonian to earliest Carboniferous resulted in the development of sedimentary basins to the west and east of Greenland and culminated in the opening of the Labrador Sea in ~ 62 Ma and the North Atlantic in ~ 56 Ma^2^. Atlantic sea floor spreading was concurrent with the passing of the Icelandic plume beneath Greenland at ~80 to 50 Ma^3,4^, resulting in the formation of Eastern and Western flood basalt provinces^2^.

Subglacial geology plays a critical role in ice flow dynamics and ice-bedrock coupling where (i) a hard bedrock can result in the overpressure of subglacial water decoupling the ice and substrate; or, (ii) soft, wet till layers reduce basal friction encouraging basal sliding^5–7^. Basal till layers are generated by the erosion of the soft rock, usually sediments, by the overlying ice^6^. For example, the fast ice streams of the West Antarctic Ice Sheet are typically underlain by wet till and sedimentary basins hundreds of metres to kilometres thick^5,6,8–11^. Borehole and seismic surveys have been used to infer the basal conditions at various locations along the west of Greenland. Till layers have been identified beneath the ablation zone^12–14^, which are accompanied by sedimentary rock layers on the order of tens to a hundred metres^15^. In addition, till has also been observed beneath the North-East Greenland Ice Stream, Greenland’s only ice stream, but the sedimentary source rocks have yet to be identified^16^. However, Harper et al.^17^ drilled 32 boreholes in the Kangerlussuaq region of Greenland and identified predominantly hard bed conditions. Where any till was observed, it was entrained in the ice and limited to a few decimetres^17^.

In addition to the mechanical properties of the basal substrate, the thermal condition of the subglacial environment plays a fundamental role in the generation of water from geothermal and frictional heating of the ice from beneath^18^. Fast ice flow and wet subglacial basal conditions are concurrent with regions of high geothermal heat flux^19–22^. These regions of elevated geothermal heat flux beneath the ice sheet are the result of the Iceland plume, which traversed the island ~80 to 50 Ma^3,4,19,20,23^. Several hotspot tracks have been proposed based on tectonic reconstructions, mantle dynamics and rock outcrops on both east and west coasts, but differ significantly with increasing age towards the west^3^. Constraining the effect of the Icelandic plume track is therefore important in testing model simulations of the Greenland Ice Sheet.

Until the early 2000s the study of the crustal structure of Greenland had been mostly limited to coastal regions due to the inaccessibility of Greenland’s interior^24^. However, a series of temporary seismic networks were deployed from the turn of the century and included both on and off-ice stations, culminating in the permanent installation of the Greenland Ice Sheet Monitoring Network (GLISN)^25^ in 2009. Receiver functions have been used to characterise the Moho depth ( ~ 40–50 km), which displayed little lateral variation and is consistent with cratonic regions^26,27^. Walter et al.^15^ used receiver functions from teleseismic events to identify sedimentary rock layers up to ~ 160 m thick beneath the ablation zone of West Greenland. Tomographic imaging using both regional and global earthquake data^24,28–32^, as well as ambient noise data^33,34^ have been used to image the lithospheric structure and potential Icelandic hotspot track beneath the GrIS and wider Arctic. Unfortunately, the resolution of these seismic studies has been limited to depths of > 5 km due to a lack of local seismicity and the large station distances^29^, and so offer limited insight into the subglacial conditions beneath the GrIS.

In this study, we measure and invert Rayleigh wave ellipticity measurements (the horizontal-to-vertical ratio of Rayleigh wave particle motions) for the 1-D shear wave velocity (*V*_*s*_) structure of the upper 5 km beneath the Greenland ice sheet. Inverting Rayleigh wave ellipticity is particularly well suited to determining the crustal structure beneath the seismic stations in regions of uneven or sparse station coverage^35^. In particular, there are many methods available to measure horizontal-to-vertical ratio and ellipticity from seismic ambient noise^36–38^. In this study, we use the degree-of-polarisation-ellipticity (DOP-E) method^39^, which allows the extraction of short period Rayleigh wave ellipticity from ambient noise measurements and enables characterisation of the upper 5 km of the crust. To our knowledge this is the first attempt to characterise the upper crustal structure of Greenland.

## Results

### Rayleigh wave ellipticity

**Fig. 2:**
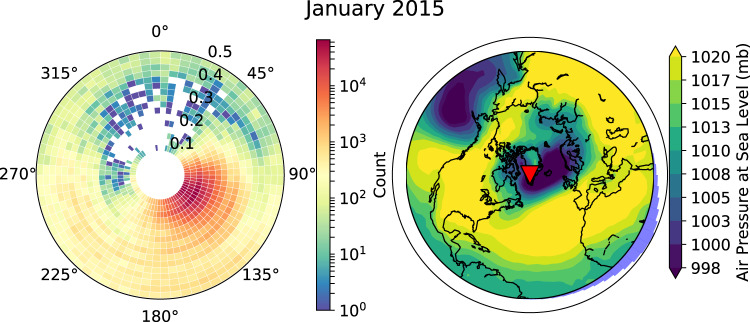
Comparison of polarisation direction of measured Rayleigh waves and air pressure at sea level for January 2015 measured at station DY2G. Left: Polar histogram of polarisation directions as a function of frequency in Hz of measured Rayleigh wave with a DOP ≥ 0.95. Right: Map of monthly average air pressure at sea level, which acts as a proxy for ocean wave interactions. The histogram along the edge of the map is the sum of all polarisation directions of measured Rayleigh waves and the red triangle is the location of station DY2G.

We compiled annual estimates of period-dependent Rayleigh wave polarisation and ellipticity from 2012 to 2017 (for full details of the technique, see the Methods section). The main source region of Rayleigh waves is the Denmark Strait in the north Atlantic between Greenland and Iceland (Fig. 2) and is dominated by periods between 5 and 10 s. These observations are consistent with regions of low air pressure, which are associated with the generation of secondary microseisms^40^.

From late April to September we observe a reduction in the number of sources originating in the Denmark Strait with secondary sources of Rayleigh waves observed at periods shorter than 4 s (Supplementary Fig. 1). Stations on the west coast of Greenland show polarisation directions from the south-west and west with a northern migration in source location along the Labrador Sea as the summer progresses. This seasonal migration in Rayleigh wave source throughout the year is consistent with the findings of Sergeant et al.^40^. Stations on the north-east coast of Greenland however show Rayleigh waves originating from the Svalbard region in the Arctic Ocean.

**Fig. 3:**
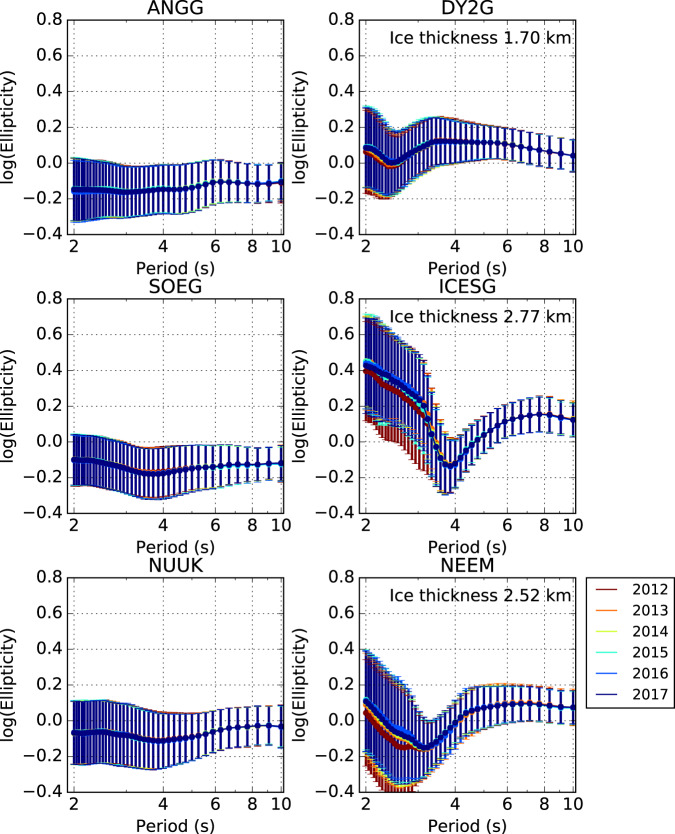
Comparison of annual ellipticity measurements with a DOP ≥ 0.95 from 2012 to 2017. Left: Illustrative examples of off-ice stations. Right: Illustrative examples of on-ice stations.

The annual ellipticity results show minimal variation in measurements and uncertainty from year to year (Fig. 3). The annual ellipticity curves of the on-ice stations show an inflection point in the shorter period data (*T* ~ 3–4 s), which is dependent on ice thickness (Fig. 3). Uncertainties of the ellipticity measurements for on-ice stations are larger than for their off-ice counterparts, with short period data having larger errors. It can be clearly seen that the log of the ellipticity follows a Gaussian distribution, with the shorter period measurements having a much broader distribution than their longer period counterparts (Supplementary Fig. 2).

**Fig. 4:**
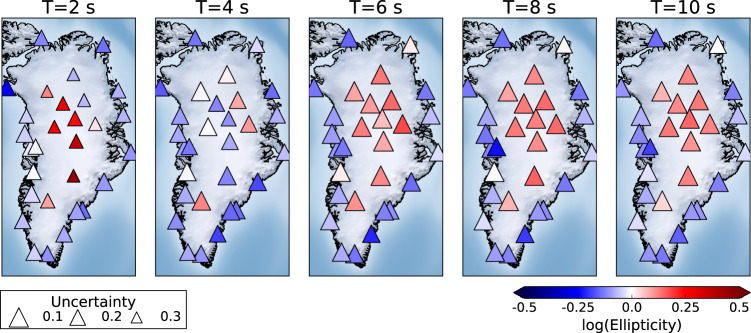
Summary of ellipticity measurements for all stations as a function of period in 2015 with a DOP ≥ 0.95. The colours represent the ellipticity while the size of the triangle represents the uncertainty of each measurement.

Figure 4 depicts the spatial variation in annual ellipticity calculated as a function of period for 2015. We select 2015 since it has the maximum number of deployed broadband seismometers in the period 2012–2017. It can be clearly seen that the off-ice stations have a negative log ellipticity for all periods while the on-ice stations show greater variation moving from positive at *T* ~ 2s, negative for *T* ~ 4s and returning to positive values for other periods up to 10s. These geographical differences highlight the importance of ice on ellipticity and generalise the observations shown in Fig. 3.

### Crustal velocity model inversion

We apply the inversion scheme explained in the Methods section to data from each station to generate 1-D *V*_*s*_ profiles. Following the results from the synthetic tests presented in the previous section, the ellipticity measurements are filtered using their standard deviation (SD), with measurements with SD > 0.2 being rejected.

**Fig. 5:**
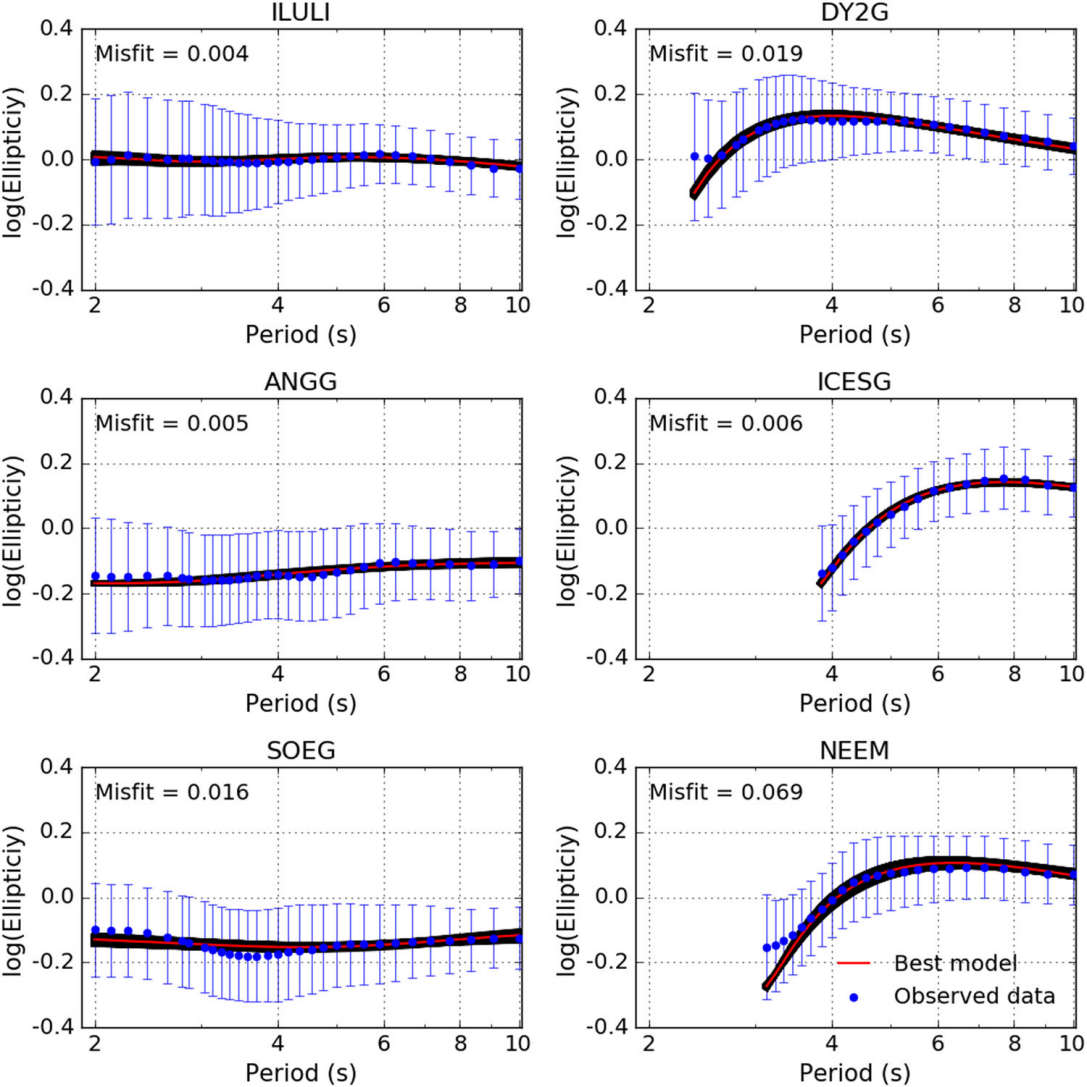
Comparison of ellipticity measurements and fundamental mode predictions from the best-fitting *V*_*s*_ models for illustrative off- (left column) and on-ice (right column) stations. The measured ellipticity (blue dots), standard deviation (error bars), predictions for the models within the uncertainty range (black lines) and the minimum misfit model (red line) are all shown.

**Fig. 6:**
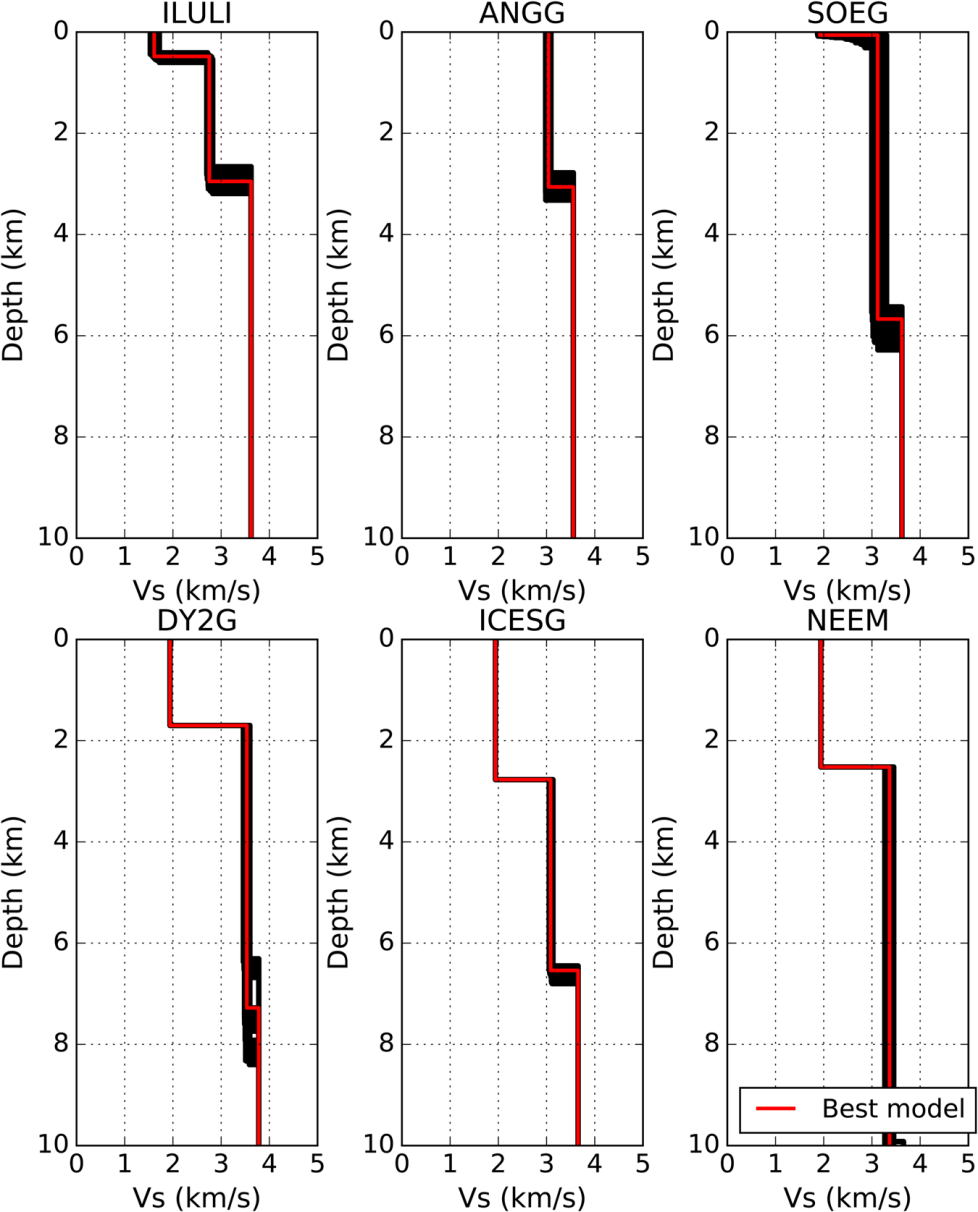
1-D *V*_*s*_ profiles computed for illustrative off-ice (top row) and on-ice (bottom row) stations. Ensemble models defining the uncertainty ranges are selected as those models that are within 20% of the minimum misfit. The red line is the minimum misfit model and the black lines are ensemble models.

We observe a good agreement between the measured ellipticity and the predictions from the formally best-fitting models (Fig. 5 and Supplementary Fig. 3). The 1-D *V*_*s*_ profiles show little variance in *V*_*s*_ with the layer thickness showing the greatest uncertainty (Fig. 6 and Supplementary Figs. 4 and 5), similar to our findings from synthetic inversion tests (see Methods). Moreover, we do not observe a trade-off between *V*_*s*_ and layer thickness in the inversion of ellipticity data (Supplementary Fig. 6)^41^.

Further, we use independent datasets to validate our crustal models. First, we model seismic waveforms from a Mw 4.6 earthquake that occurred on the southern tip of Greenland on the 11th of April 2013 and compare them with recorded data. We employ a normal mode summation approach^42^ to compute the synthetic waveforms using the GCMT source model and this study’s 1-D crustal model. Synthetic waveforms computed using our 1-D crustal model closely match the observed waveforms and improve the data fit compared to synthetics computed for the LITHO1.0 model (Supplementary Figs. 7 and 8). We note that there is some discrepancy between the observed and synthetic waveforms at NEEM, which is the furthest station from the earthquake. This is likely due to deviations between the crustal structure of the full earthquake travel-path and our local 1-D model as well as uncertainties in the source mechanism. Secondly, we compare fundamental mode group velocity calculations using our new crustal models beneath on-ice stations DY2G, ICESG, SUMG and NEEM with previously published measurements derived from ambient noise cross correlation^33,43^(Supplementary Fig. 9) . The results show good agreement between our calculated group velocities and the measurements^33,43^.

**Fig. 7:**
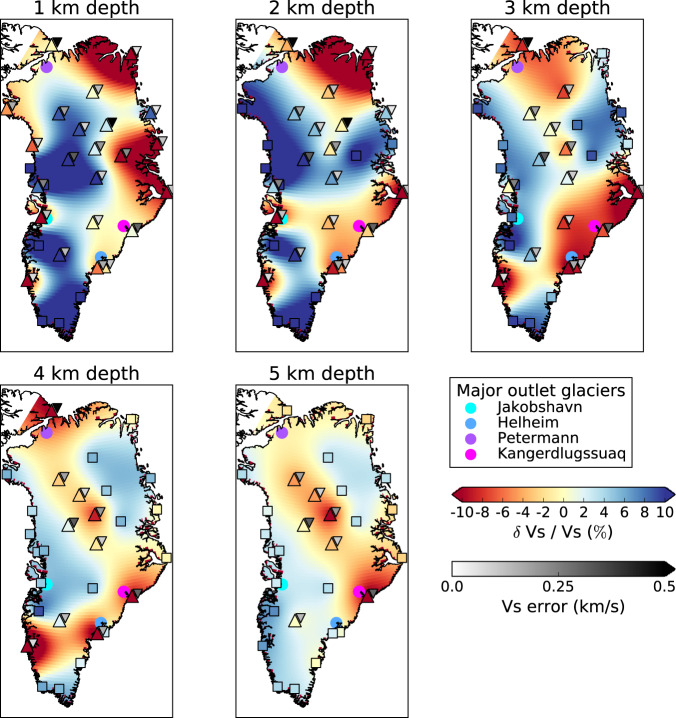
Percentage perturbation shear wave speed at each station with respect to the average at each depth slice. The colours of the upright triangles are the *δV*_*s*_ percentage perturbation values calculated at that station. The squares are stations that match LITHO1.0. The smaller inverted triangles are the range of *V*_*s*_ taken from the 20% ensemble models. For on-ice stations, the ice-sheet layer has been removed.

The 1-D *V*_*s*_ profiles are interpolated at each 1 km depth interval onto a 0.25^∘^ × 0.25^∘^ uniform 2-D grid. Following the ellipticity studies of Attanayake et al.^44^ and Berbellini et al.^45^ we apply Kriging interpolation, a technique often used for spatial geographical interpolation of sparse or irregularly sampled data, which has the favourable statistical property of estimating the best linear unbiased prediction at the unsampled locations^46,47^. We note that uncertainties in the interpolated values increase with distance from the seismic stations (Supplementary Fig. 10). Prior to interpolation at on-ice stations the ice layer is removed such that the ice-bedrock interface is classified as the surface, allowing for direct comparison of the upper-crust with results from off-ice stations. Figure 7 illustrates the geographical distribution of the percentage deviation of *V*_*s*_ relative to the average at 1 to 5 km depths, which we shall refer to as *δV*_*s*_.

At 1 km depth the map is dominated by strongly contrasting regions of higher and lower *δV*_*s*_ relative to the average measured at each station. The western interior of Greenland is characterised by high *δV*_*s*_ with a transition to lower velocities seen along a north-west to south-east oriented boundary running through Greenland’s interior. The north and east coastal regions are dominated by low *δV*_*s*_ concurrent with sedimentary outcrops in the region. These low *δV*_*s*_ regions encroach into the interior of the ice sheet synchronous with the North-East Greenland Ice Stream. Low *δV*_*s*_ regions are seen around the major outlet glaciers of Jakobshavn, Helheim, Petermann and Kangerdlugssuaq.

In the 2 km depth slice a number of the shallow low-velocity features along the north-west and east coasts decrease and now match the LITHO1.0 model (squares Fig. 7). However, a number of features present at 1 km depth remain at this depth, such as, e.g., a low *δV*_*s*_ anomaly in the north and the east of Greenland. In addition, weak anomalies that are present at 1 km depth become more prominent at 2 km, namely the linear north-west to south-east, ~ 1 to 2 %*δV*_*s*_ anomaly cutting through the centre of Greenland. The low *δV*_*s*_ anomalies at the outlet glaciers also become more prominent at this depth.

At 3 km depth the majority of stations along the west and north-east coast of Greenland now match the LITHO1.0 model (squares Fig. 7). Stations that do not match LITHO1.0 all exhibit negative *δV*_*s*_ of ~ –2 to –4 % and are confined to the linear north-west to south-east trending feature through the centre of Greenland and the south-east and northern coast. The low-velocity region from SUMG to the coast and around Helheim and Kangerdlugssuaq is further highlighted and corresponds to the catchment area of these glaciers.

**Fig. 8:**
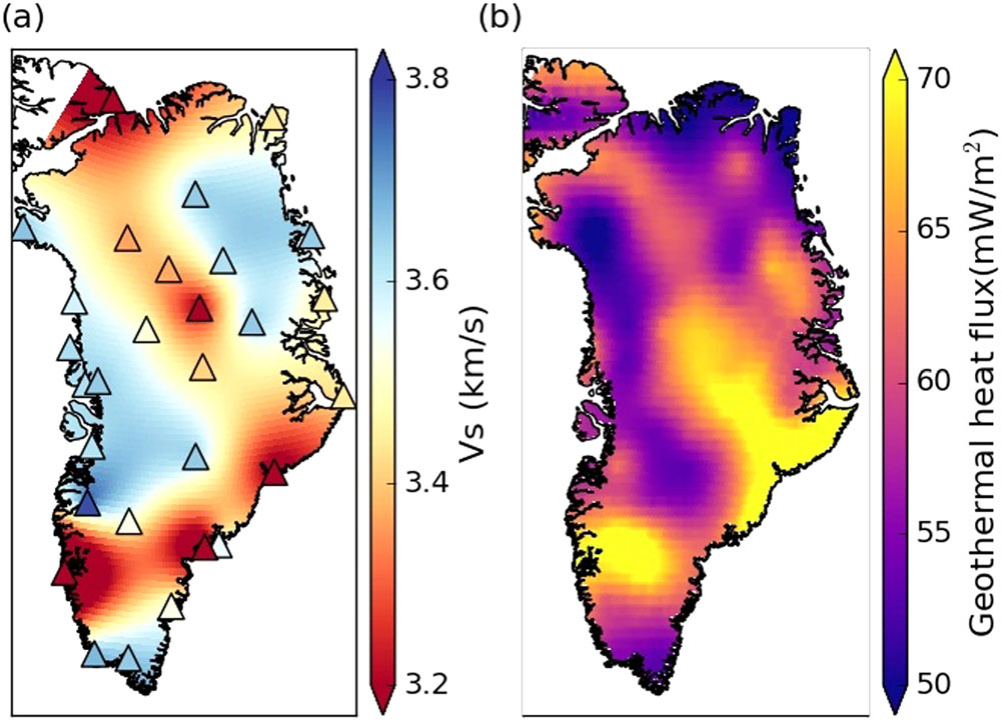
Comparison between *V*_*s*_ and geothermal heat flux model. **a** *V*_*s*_ at 4 km depth calculated at each seismic station (triangle) and interpolated onto a 0.25^∘^ × 0.25^∘^ grid. **b** Geothermal heat flux at the ice-bedrock interface taken from Martos et al.^3^.

Both the 4 and 5 km depth slices display similar characteristics, with the vast majority of stations now matching the LITHO1.0 model (squares Fig. 7). The dominant feature at these depths is the linear north-west to south-east trending feature through the centre of Greenland (Fig. 7). The location of these deeper, negative *δV*_*s*_ regions are concurrent with locations of high geothermal flux and igneous intrusions (Fig. 8) in central and northern Greenland.

## Discussion

Our analysis highlights a heterogeneous crust beneath Greenland. The range of our calculated shear wave velocities *V*_*s*_ is similar to those estimated in the tomographic images at 10 km depth^24^. The North-East Greenland Ice Stream (NEGIS), Greenland’s only ice stream, discharges more than 10% of the Greenland Ice Sheets area with fast velocities observed up to the ice divide^48^. Major outlet glaciers Jakobshavn, Helheim and Kangerdlugssuaq, which are located on the central east and west coasts, are some of the fastest-flowing glaciers in the world, together draining ~15.6% of the Greenland Ice Sheet area^49^.

Jakobshavn Isbræ is a marine-terminating glacier that drains into the Ilulissat Icefjord located at Disco Bay on the central west coast of Greenland (Fig. 7). Disco Bay defines the northern extent of the Nagssugtoqidian mobile belt and the on-shore geology primarily comprises a Precambrian crystalline basement of reworked gneisses, granites and metavolcanic and metasedimentary rocks (Fig. 1)^2,50–52^. Disco Bay comprises Upper Cretaceous and Palaeogene sediments with thickness on the order of 100s of metres to 1–2 km draped above the Precambrian basement^50–52^. Our 1-D *V*_*s*_ profiles obtained for station ILULI, located close to the town of Ilulissat, comprise two layers: a shallow 480 ± 190 m thick layer with *V*_*s* _= 1.6 ± 0.19 km s^−1^, which overlies a 2.95 ± 0.53 km thick layer with *V*_*s*_ = 2.95 ± 0.12 km s^−1^ (Figs. 1, 5, and 6). We interpret the first layer as Pre-Quaternary sediments based on the correspondence of our estimated *V*_*p*_ = 3.14 km s^−1^ with the P-wave interval velocity (*V*_*p*_ = 3.2 km s^−1^) used by Chalmers et al.^50^. The classification of the second layer (*V*_*p* _= 4.65 km s^−1^) is more challenging, with both basalts and Precambrian basement rocks being suitable candidates based on the local geology and seismic velocities (*V*_*p*_ = 4.5 km s^−1^)^50^. Previous reflection seismic studies^50^ have been unable to distinguish between these two rock types. We note that this layer is seismically slower (*δV*_*s*_ ~ –2%) at depths of up to 3km relative to other stations on the West of Greenland, making the interpretation difficult (Fig. 7). We speculate that this layer is a mechanically weakened and reworked Precambrian crystalline basement, as evidenced by the erosion of a deep trough several hundred metres to a kilometre deep beneath Jakobshavn Isbræ^12^ and the presence of sedimentary layers^15^.

Helheim and Kangerdlugssuag glaciers are large tidewater glaciers on the south-east coast of Greenland that terminate in the Sermilik and Kangerdlugssuag Fjords, respectively. Stations ISOG and ANGG are located on either side of the mouth of Sermilik Fjord, while station SOEG is located at the mouth of Kangerdlugssuag Fjord (Fig. 1). The geology of south-east Greenland is predominantly reworked Archaean gneiss with interbedded metasediments from the early Proterozoic and part of the Nagssugtoqidian mobile belt^2^ (Fig. 1). SOEG is located close to Tertiary magmatic intrusions generated by the continental breakup of the North Atlantic.

The models obtained for stations ANGG, ISOG and SOEG show negative *δV*_*s*_ anomalies of ~ -2 to -6%, which are depth dependent and reach a maximum at about 3 km depth. For station ANGG, we obtained a model with a single layer with thickness of 3.06 ± 0.54 km and a *V*_*s*_ = 3.04 ± 0.11 km s^−1^, while for ISOG we obtained a double layer with thicknesses of 0.05 ± 0.03 km and 4.60 ± 0.35 km, and velocities of 0.84 ± 0.66 km s^−1^ and 2.91 ± 0.16 km s^−1^ respectively. For station SOEG, we again obtained two layers; the first layer had a thickness of 0.06 ± 0.27 km and *V*_*s*_ = 1.91 ± 1.01 km s^−1^, and the second layer had a thickness of 5.61 ± 0.85 and *V*_*s*_ = 3.12 ± 0.3 km s^−1^ (Figs. 5 and 6). Given the broad similarities between the results for stations ISOG and SOEG, we interpret the first layer beneath these stations as a near surface layer including sediment, with the second layer having a similar composition to that beneath the ANGG station based on layer thickness and *V*_*s*_. These latter layers are interpreted as reworked gneiss from the Ammassalik mobile belt. These deformed rocks are consistent with hypothesis that metamorphic rocks act as hydraulic pathways for the discharge of large volumes of freshwater into the sea along the SE coast of Greenland^53^. It has also been suggested that the Ammassalik mobile belt is an extension of Nagssugtoqidian mobile belt on the west coast^2^, which is supported by the similarity of *V*_*s*_ and layer thicknesses at ILULI on the west coast and ISOG, ANGG and SOEG on the east. This hypothesis is affirmed by the results obtained for the on-ice station ICESG, which lies on the ice divide between the Ammassalik and Nagssugtoqidian mobile belts. It has a sub-ice layer with thickness 3.77 ± 0.35 km and *V*_*s*_ = 3.09 ± 0.08 km s^−1^, similar to the stations on the east and west coasts.

The majority of stations along the west coast and penetrating into the interior of Greenland (DY2G and NE2) show positive *δV*_*s*_ anomalies, which indicate the presence of hard bedrock. Additionally, we interpret the negative *δV*_*s*_ anomalies beneath stations ISOG, ANGG, SOEG and ICESG as reworked or damaged Precambrian rocks, which are easily eroded to produce the deep troughs at Jakobshavn, Helheim and Kangerdlugssuaq. We do not observe any thin sedimentary layers < 100 m to the west and south-central east of the Greenland Ice Sheet. However, we acknowledge that the lack of short period data and thick ice sheet limits our ability to recover thin sedimentary layers, which could be widespread beneath the ice sheet.

The north and north-east of Greenland is dominated by very low *δV*_*s*_ < -5 % up to ~3 km depth. At 1 km depth the low velocities along the east coast closely match the Caledonian fold belt (Fig. 7). The Caledonian fold belt comprises early Proterozoic gneisses and granitoid basement, which are overlaid by Proterozoic and Palaeozoic sediments prior to the orogeny^2^. We attribute the lower *V*_*s*_ to the presence of the sedimentary units in the fold belt. In the north, the inversion of data from station NOR led to a 2.13 ± 0.11 km thick layer with *V*_*s*_ = 2.0 ± 0.05 km s^−1^, which is consistent with the presence of large Palaeozoic sedimentary basins in the region (Fig. 7).

Ice-penetrating radar measurements have identified extensive regions along the coast and ice divide where basal melt is present and attributed to the onset of fast ice flow of the NEGIS^19,20,22,54^. In addition, the refreezing of basal meltwater alters the stratigraphy, rheology and temperature structure of the ice, enhancing its ability to deform and flow^54^). Bell et al.^54^ identified large units several hundred metres thick of warm, soft ice produced by the refreezing of basal water and attributed to the onset of fast ice flow at the Petermann Glacier. Along the ice divide of the GrIS, geothermal heat flux plays a leading role in the generation of basal melt^18^. The source of this elevated heat flux is believed to be related to the Icelandic mantle plume that traversed beneath Greenland ~80 to 50 Ma^3,4,20^. Although a variety of plume and geothermal heat flux models have been proposed, the thermal effect on the ice-sheet bed has so far remained poorly constrained due to limited borehole measurements and significant discrepancies between models derived from seismic^4,33,55^ and magnetic^3^ data. Seismic derived geothermal heat flux models use tomographic models of the crust and upper mantle, correlating regions of low *V*_*s*_ with regions of elevated heat flux^4,33,55^. The location of these low *V*_*s*_ regions varies between models, with robust measurements limited to depths greater than 10 km^24,32,33^. Magnetic derived geothermal heat flux models are also depth limited because airborne or satellite data are used to compute the Curie temperature to constrain the model^3,56^. Both types of models are therefore reliant on observations at depth to simulate the geothermal heat distribution at the ice-bedrock interface. These uncertainties in the geothermal heat flux models have been shown to have a significant effect on ice-sheet models, with NEGIS discharge uncertainties of 2.10 Gt per yr^57^. Finally, topography has also been shown to play a role in the distribution of heat flow particularly at the scale of individual glaciers and catchments^58^.

At 4 and 5 km depth our model shows strong spatial consistency between regions of *V*_*s*_ < 3.4 km s^−1^ and regions with elevated geothermal heat flux ( > 60 mW m^−2^) in the model of Martos et al.^3^. In particular, the south-east to north-west linear low *V*_*s*_ feature that traverses Greenland can be clearly seen in the geothermal heat flux model (Figs. 7 and 8). We also observe that the low *V*_*s*_ found beneath NUUK (Fig. 1) up to 4 km depth correlates with a region of high heat flux^3^ (Fig. 7). Regions of basal melt derived from airborne radar data^22^ show a strong degree of spatial consistency with regions of high geothermal heat flux^3^. In particular, regions around station NE1 (Fig. 1) show a concentration of basal melt inferred from radar data^22^, whereas boreholes drilled at NEEM and GRIP (SUMG) have identified a frozen bed. However, ice cores from NEEM found inverted ice strata with old ice from the Last Glacial Maximum, which is typically found at the ice-sheet bed, close to the ice surface^59^. Ice from the surrounding area of the drill site also displays evidence of enhanced deformation^54^. Moreover, studies of ice structure at SUMG identified strong seismic anisotropy generated by the partial melting and deformation and alignment of ice crystals^60,61^. The low *V*_*s*_ in the region at depths of 2 km in the crust (Figs. 7 and 8) is interpreted as evidence of geothermal heat flux that leads to the formation of warm deformable ice through the melting and refreezing of meltwater, which is then responsible for the onset of fast ice flow at Petermann Glacier^54^. We also attribute the low *V*_*s*_ at NE1 and the concentration of basal melt water in the region^22^ as being due to the elevated geothermal heat responsible for the onset of the NEGIS, and is consistent with previous studies^19–21,62^. Therefore, we attribute the source of the *V*_*s*_ anomaly through the centre of Greenland as the surface expression of the Iceland hot spot track beneath Greenland. Results from on-ice stations NEEM, NE1, NE3 and SUMG closely match the hotspot track suggested by Martos et al.^3^.

Stations NE4 and NE6 are located within the catchment of the NEGIS. The single layer models obtained for stations NE4 and NE6 show layer thicknesses of 1.75 ± 1.25 km and 3.56 ± 0.61 km and low *V*_*s*_ of 2.9 ± 0.15 km s^−1^ and 3.1 ± 0.12 km s^−1^, respectively, relative to the background average. We interpret these relatively low seismic velocities at NE4 and NE6 as the source of the till identified along the North-East Greenland Ice Stream from controlled-source reflection seismic surveys^16^. In the absence of a more compelling alternative hypothesis we speculate that the source rocks of the till layers along the NEGIS are the product of geothermally altered or weakened basement rocks, which are readily eroded, facilitating fast ice flow.

Our seismic model therefore provides unique and new information on the uppermost crustal geology of Greenland and its implied influence on ice dynamics. The identification of slow seismic velocities at the fast outlet glaciers of Jakobshavn, Helheim and Kangerdlugssuaq are interpreted as the signature of weak or damaged crustal rocks that are easily eroded to produced soft deformable till, which facilitates this fast ice flow. These damaged rocks at Helheim and Kangerdlugssuaq support the hypothesis that these rocks act as hydraulic pathways and reservoirs for basal ice melt discharging large volumes of freshwater along the SE coast^53^. We also identify the subglacial expression of the Iceland hotspot track that traversed Greenland ~ 80 to 50 Ma, which controls the onset of fast ice flow of Petermann Glacier and NEGIS. Our model provides much needed constraints on the geothermal heat flux estimates, which have been shown to have a significant effect on ice-sheet models resulting in discharge uncertainties of 2.10 Gt per yr for NEGIS^57^. Moreover, we observe geothermally weakened basement rocks, producing soft till which facilitates fast ice flow beneath the NEGIS. Asserting the basal thermal conditions and their effects on the flow of the NEGIS, outlet glaciers and the dynamics of the GrIS should therefore be a priority for future in situ measurement campaigns. Numerical simulations of ice-sheet flow and sensitivity to future atmospheric and ocean warming should consider the potentially enhanced sensitivity of these outlet glaciers and their upstream ice catchments to basal thermal forcing.

## Methods

### Rayleigh wave ellipticity measurements

Rayleigh waves are surface waves with particle displacements polarised along a vertical ellipse and typically have retrograde motions at the Earth’s surface. Rayleigh wave ellipticity is the ratio of the horizontal to vertical axis of the elliptical particle motion, and under ray theory assumptions, it depends only on the subsurface directly below the station^63^. In regions of sparse or limited station coverage, ellipticity measurements provide a powerful tool for probing the subsurface structure beneath a seismic station. Recent years have seen an increasing application of earthquake-derived ellipticity measurements to generate 1-D crustal models beneath each station^35,45^. Moreover, recently Berbellini et al.^39^ developed the DOP-E method to extract ellipticity measurements from seismic ambient noise. The DOP-E method uses the polarisation to identify weak Rayleigh wave signals and subsequently compute period-dependent ellipticity^39^. The identification of Rayleigh waves is done using the degree-of-polarisation (DOP) approach^64,65^, which is briefly described below.

Three-component seismograms recorded in the north, east and vertical directions are transformed into the time-frequency domain^65^ using the S-transform, and collated in a spectral matrix. The S-transform has properties similar to the Short Term Fourier Transform, but employs a window length, which is scaled to the periods of interest permitting multiresolution signal analysis^66^. A moving window eigen-decomposition of the spectral matrix permits the calculation of the semi-major and semi-minor axes of the best-fitting ellipse to the data to be computed as a function of time and period^65,66^. The planarity vector of the signal is defined as the vector product of the semi-major and semi-minor axes and is perpendicular to the best-fitting ellipse. For elliptical signals the unit planarity vector is expected to be constant and as such is used for the definition of the DOP for Rayleigh waves^40,64–66^. DOP is calculated as the projection of the instantaneous unit planarity vector on the mean planarity vector for the data window and ranges between 0 and 1, where 0 indicates randomly changing polarisation throughout the data window and 1 is a stable polarisation measurement^39,40,65,66^. To further improve Rayleigh wave detection, the DOP estimates are down-weighted based on the deviation of the best-fitting ellipse from the vertical. Finally, once the DOP has been estimated and is above a given threshold, the instantaneous semi-major and semi-minor vectors are used to compute the period-dependent ellipticity measurements^39^.

For this study the seismic data were pre-processed to remove the instrument’s response and bandpass filtered between 0.2 and 50 s. To validate the pre-processing, the DOP-E method was applied to earthquakes with magnitudes > 8, where measured back azimuths were compared with the expected value based on the source location and great circle path (Supplementary Fig. 11). The ambient noise data were also manually inspected prior to the application of the DOP-E method to data with periods between 2 and 10 s, which are sensitive to the upper 10 km of the crust^39^. For each period only the best measurements are retained by selecting those with a DOP ≥ 0.95 (Supplementary Fig. 1). We construct ellipticity curves by summarising the distribution of measurements at each period by computing the median and uncertainties at the 13.6 and 86.4 percentile.

### Rayleight wave ellipticity inversion

We follow the ellipticity inversion scheme of Ferreira et al.^35^ and Berbellini et al.^39,45^ to generate 1-D *V*_*s*_ profiles directly beneath each station. The inversion uses the Neighbourhood Algorithm (NA)^67^, which is a self-adaptive Monte Carlo approach that efficiently samples the parameter space. The NA search is tuned by three parameters: (i) the number of random *V*_*s*_ models sampled in the first iteration; (ii) the number of models created at each iteration; and, (iii) the number of best-fitting models around which the algorithm will continue to the next iteration^67^. Having tested different parameters, we obtained stable inversion results when performing an initial search of 1000 models, followed by 20 models for each subsequent iteration where 5 best-fitting models were selected for the next iteration. The NA outputs an ensemble of models along with their data misfit that can be used to empirically assess model uncertainties. Following these previous studies, the uncertainties in *V*_*s*_ are defined by the range of models with misfit not exceeding 20%, the lowest misfit value obtained in the inversion.

Assuming that the ellipticity measurements include only fundamental mode Rayleigh waves, we compute theoretical ellipticity curves using a normal mode approach as implemented in the software package of Herrmann^42^. In addition to *V*_*s*_, the input 1-D Earth model also includes seismic P-wave velocity (*V*_*p*_) and density (*ρ*) profiles, which are estimated empirically from *V*_*s*_ using the generalised Brocher relations^68^. Finally, the misfit function *m* minimised during the inversion is given by:

1 $$m=\mathop{\sum }\limits_{i=1}^{N}\frac{{\left({d}^{i}-{g}^{i}\left({{{{{{{\bf{x}}}}}}}}\right)\right)}^{2}}{{\left({\sigma }_{D}^{i}\right)}^{2}},$$

where *d* is the measured ellipticity, *g*(**x**) is the predicted ellipticity for the sampled model **x**, *σ*_*D*_ is the uncertainty in the measurements described previously and *N* is the number of measurements. Selecting an appropriate parametrisation is fundamental to obtain an accurate representation of the subsurface. We tested different layered parametrisations in the inversions and considered models with up to 3 subsurface layers with variable thickness and *Vs* ranges. Berbellini et al.^39^ showed that ellipticity data with periods between 2 and 10 s are sensitive to the upper 10 km of the subsurface, which is corroborated by sensitivity kernels (Supplementary Fig. 12). Thus, we fix the maximum depth permitted in the inversions to not exceed the depth of the first crustal layer defined in LITHO1.0 ( ~ 10–13 km)^69^. Candidate models where the layer depths are greater than the fixed depth are truncated with the remaining model given by LITHO1.0^69^. We performed extensive tests and found that a single subsurface layer is sufficient to model the ellipticity measurements for the majority of stations, except for the off-ice stations ILULI, ISOG, TULEG and SOEG. For the latter stations we found that two subsurface layers including a top layer of ~ 500 m were needed to fit the T < 3–4 s data. In the single layer case, the layer’s thickness is allowed to vary from 0 km to the thickness of the first crustal layer in LITHO1.0^69^. *V*_*s*_ ranges between 0.2 km s^−1^ and the value given in LITHO1.0 for the first crustal layer ( ~ 3.6 km s^−1^)^69^. We found that with this *V*_*s*_ search range the inversion did not hit any bounds and hence we did not need to consider a wider *V*_*s*_ interval.

On-ice stations have an additional ice layer above the crust with a fixed ice thickness matching the Bedmachine v3 model, which is derived from a combination of airborne and satellite radar observations and mass conservation modelling^70^. The gradual accumulation and compaction of snow gives rise to changes in ice properties with depth^18^. Ice crystals are anisotropic with hexagonal symmetry, where the symmetry axis is parallel to the crystal c-axis^18^. Ice crystals are randomly orientated following deposition, which gives rise to isotropic aggregate behaviour. With increasing burial air is removed from the snow until it becomes ice, this transition stage between snow and ice is known as the firn layer^18^. The depth and properties of the firn layer is highly variable (e.g., water content, ablation rate) with ~ 80 m being the upper firn thickness observed in Greenland along the North-East Greenland Ice Stream^71^. As the ice continues to undergo burial, the c-axis of the individual crystals rotate vertically towards the direction of maximum compressive stress, giving rise to anisotropic behaviour. Seismically this manifests as VTI at depths > 1.7 km on the Greenland Ice Sheet^61^. We find that ellipticity measurements calculated for ice models with VTI at depths > 1.7 km do not show a significant difference from the isotropic ice (Supplementary Fig. 13). However, the inclusion of a firn layer leads to a more pronounced variation in ellipticity at periods sensitive to the ice < 4 s (Supplementary Figs. 14, 15). Nonetheless these differences in ellipticity are within the measurement error. Owing to the variability and poorly known extent of the firn layer thickness and minimal effect of anisotropy, we consider the ice to be a single isotropic layer with *V*_*s*_ = 1.94 km s^−1^, *V*_*p* _= 3.8 km s^−1^^72^ and *ρ* = 9.2 kg m^−3^^14^ in the ice layer, which are generally accepted values for ice on the Greenland Ice Sheet.

### Synthetic inversion tests

**Fig. 9:**
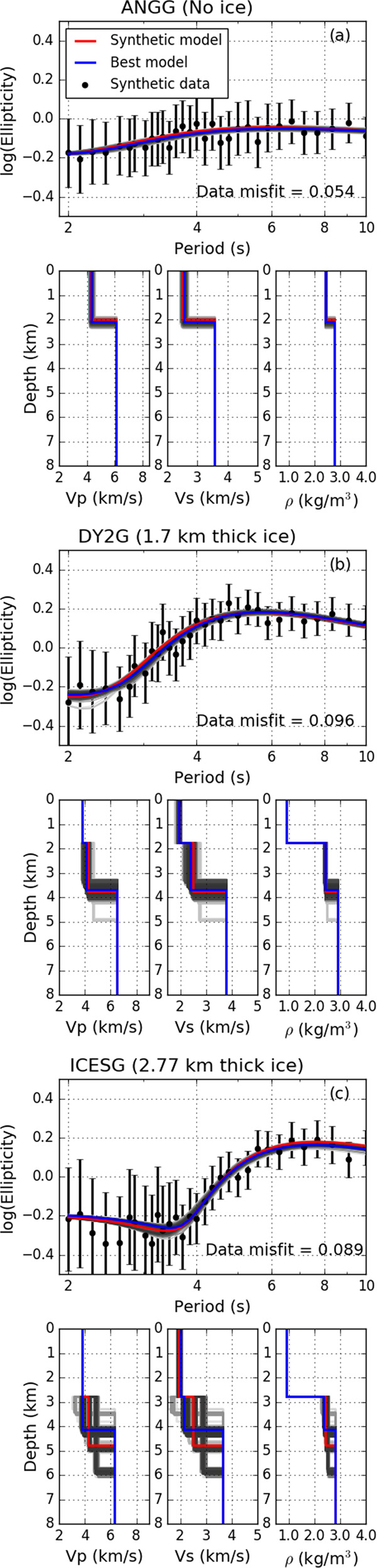
Results from synthetic velocity model inversion tests for a 2 km subsurface layer. For each station (**a** ANGG, **b** DY2G and **c** ICESG) the top panel compares the perturbed ellipticity data and fundamental model predictions for the true and the best-fitting *V*_*s*_ models. The bottom three panels are 1-D depth profiles of *V*_*p*_ (left pane;), *V*_*s*_ (middle panel) and density (right panel). In all panels the blue line is the model with the minimum misfit, the red line is the true model and the grey lines correspond to the solutions from the inversions with data misfit values within 20% of the formally best-fitting model. The black dots and error bars in the top panel are the perturbed input data.

In order to validate our proposed inversion scheme we perform a series of synthetic tests to quantify the recoverability of subsurface layer thickness and *V*_*s*_ in both off- and on-ice cases. We select three different stations for the inversion tests, which represent off-ice (ANGG), intermediate ice-thickness of 1.7 km (DY2G) and thick ice of 2.77 km (ICESG) conditions. The subsurface layer to be recovered has *V*_*s*_ = 2.5 km s^−1^ and *V*_*p*_ and *ρ* estimated from *V*_*s*_ by the Brocher relations^68^ (Fig. 9). Theoretical fundamental modes of Rayleigh wave ellipticity are computed for each candidate subsurface model^42^. Each ellipticity measurement is perturbed by computing 100 random samples from a Gaussian distribution with a mean given by the theoretical ellipticity and a standard deviation given by the uncertainty estimates for that station. The median value is then calculated from these 100 random samples (Fig. 9).

We begin by considering the off-ice case (station ANGG) for which we model a single 2 km thick layer below the surface. Figure 9 (a) shows that the results obtained from the inversion agree well with the true input subsurface model. For the on-ice cases of DY2G (1.70 km thick ice) and ICESG (2.77 km thick ice) we consider three different subsurface models with sub-ice layers with variable thickness: 1, 2, and 5 km thickness. We find that in all cases the properties of the sub-ice layer are reasonably well recovered, although we note that the depth estimates show the largest variance.

Comparing the synthetic ellipticity curves (Fig. 9) with the ellipticity measurements (Fig. 3) from the on-ice stations, we observe a clear deviation of the real data from the theoretical predictions for the wave periods that are most sensitive to the ice layer, typically *T* < 4 s. Concurrent with these deviations from the predictions, the standard deviation of the ellipticity measurements also increases, which is not observed at off-ice stations. These deviations may be due to a number of effects such as, e.g., firn structure or overtone contamination, which will be investigated in detail in future work. In order to circumvent this issue, our final synthetic inversion tests examine the capability of accurately recovering the subsurface layer by excluding ellipticity measurements, which deviate strongly from the theoretical predictions. We classify as anomalous measurements those with standard deviations > 0.2 and that show a substantial deviation from synthetic ellipticity curves (typically on-ice measurements for *T* < 3–4 s). We find that by excluding these anomalous measurements we can still recover the subsurface structure well (Supplementary Figs. 16, 17 and Supplementary Table 1), as well as mitigate variation in the near surface firn layer. Thus, these synthetic inversion tests demonstrate that our inversion scheme is capable of accurately recovering the subglacial layer beneath the ice sheet. We note that while *V*_*s*_ is overall well determined, the layer depth estimates show some variability (Supplementary Figs. 16 and 17).

## Supplementary information

Supplementary Information

## Data Availability

The seismic data were acquired and distributed by the Greenland Ice Sheet Monitoring Network (GLISN) federation and its members. The facilities of IRIS Data Services, and specifically the IRIS Data Management Center, were used for access to waveforms, related metadata, and/or derived products used in this study. IRIS Data Services are funded through the Seismological Facilities for the Advancement of Geoscience (SAGE) Award of the National Science Foundation under Cooperative Support Agreement EAR-1851048. Sea pressure were downloaded as part of the NCEP Reanalysis data from NOAA/ESRL Physical Sciences Laboratory, Boulder Colorado from their Web site at http://psl.noaa.gov/^75^.

## Code availability

The DOP-E processing code was downloaded and distributed from: github.com/berbellini/DOP-E.
