# Supplementary material for: Uppermost crustal structure regulates the flow of the Greenland Ice Sheet
Source: Nat Commun. 2021 Dec 15;12:7307. doi: 10.1038/s41467-021-27537-5 (PMC8674248; doi:10.1038/s41467-021-27537-5)
Supplement: Supplementary file 1 — Supplementary Information [file 41467_2021_27537_MOESM1_ESM.pdf]

Supplementary material: Uppermost crustal structure regulates the flow of the Greenland Ice Sheet

G. A. Jones, A. M. G. Ferreira, B. Kulessa, M. Schimmel,
A. Berbellini & A. Morelli

Table 1: Results from synthetic inversion test for on-ice stations DY2G and ICESG. Test 1 is the experiment computed with variable V_s ice layer, in Test 2 ice V_s was fixed and Test 3 represent experiment with fixed ice V_s and the ellipticity measurements filtered based on their standard deviation.

Station	Test 1 Z (%)	Test 2 Z (%)	Test 3 Z (%)	Test 1 V_s (%)	Test 2 V_s (%)	Test 3 V_s (%)
DY2G 1 km	109.0	31.0	30	27.5	13.2	12.7
DY2G 2 km	-3.5	-1.5	2.0	-4.4	-3.4	-3.6
DY2G 5 km	1.2	1.2	1.2	-2.0	-2.1	-2.2
ICESG 1 km	-27	-16.0	-15.0	-3.4	-2.3	-2.2
ICESG 2 km	-31.5	27.0	26.0	-14.3	8.4	8.2
ICESG 5 km	-2.6	-2.6	-3.4	-2.5	-2.4	-2.6

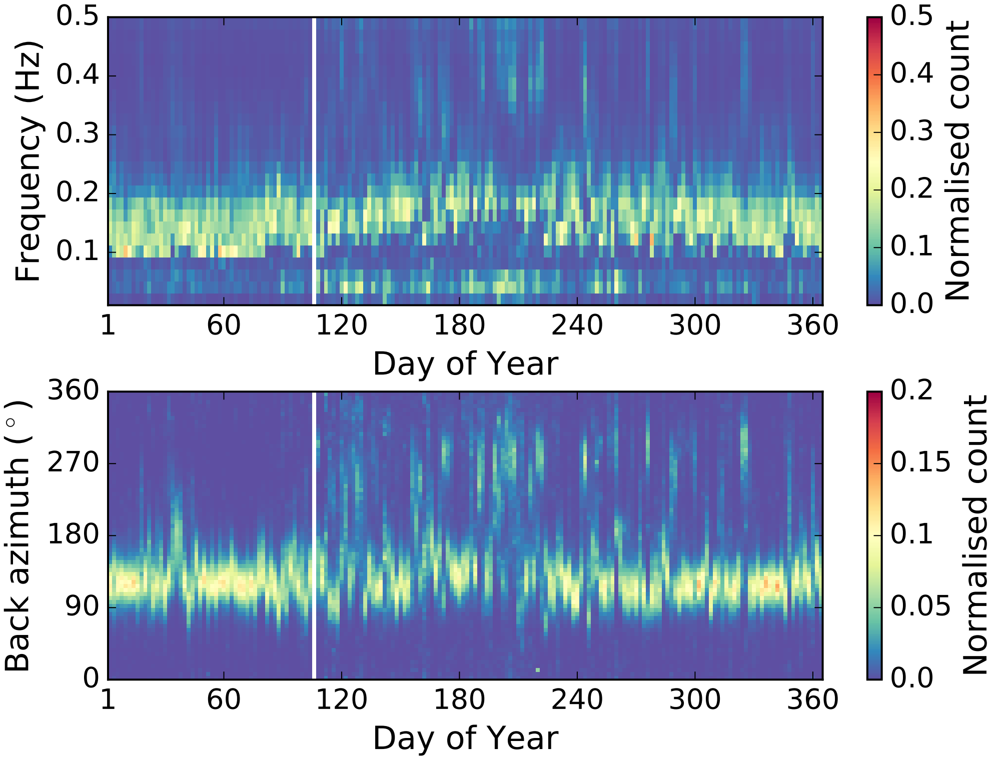

Figure 1: Comparison of frequency content (top) and source-receiver back azimuth (bottom) as a function of day of year for DY2G with a DOP of 0.95. The vertical white lines correspond to a 2 day period where data was unavailable.

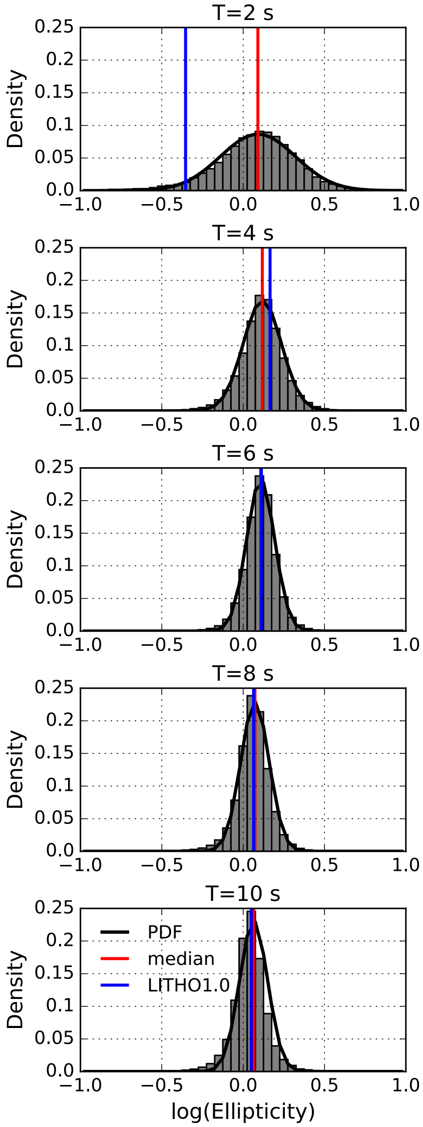

Figure 2: Representative distribution of ellipticity measurements from 2015 for on-ice station DY2G. We see that the ellipticity measurements follow a log-normal distribution across all periods with the short periods having a wider distribution. The red vertical line is the median value, the blue vertical line is the ellipticity predicted for the model LITHO1.0 (1) and the black curved line is the best fitting Gaussian distribution based on the measurements' median and standard deviation.

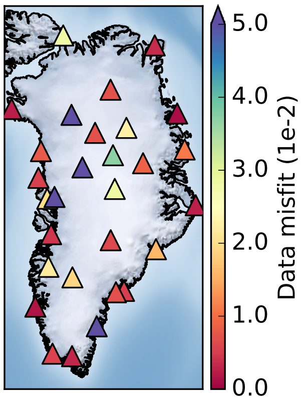

Figure 3: Summary of misfit between predictions from our 1-D crustal models and data. The colours of the triangles represent the data misfit.

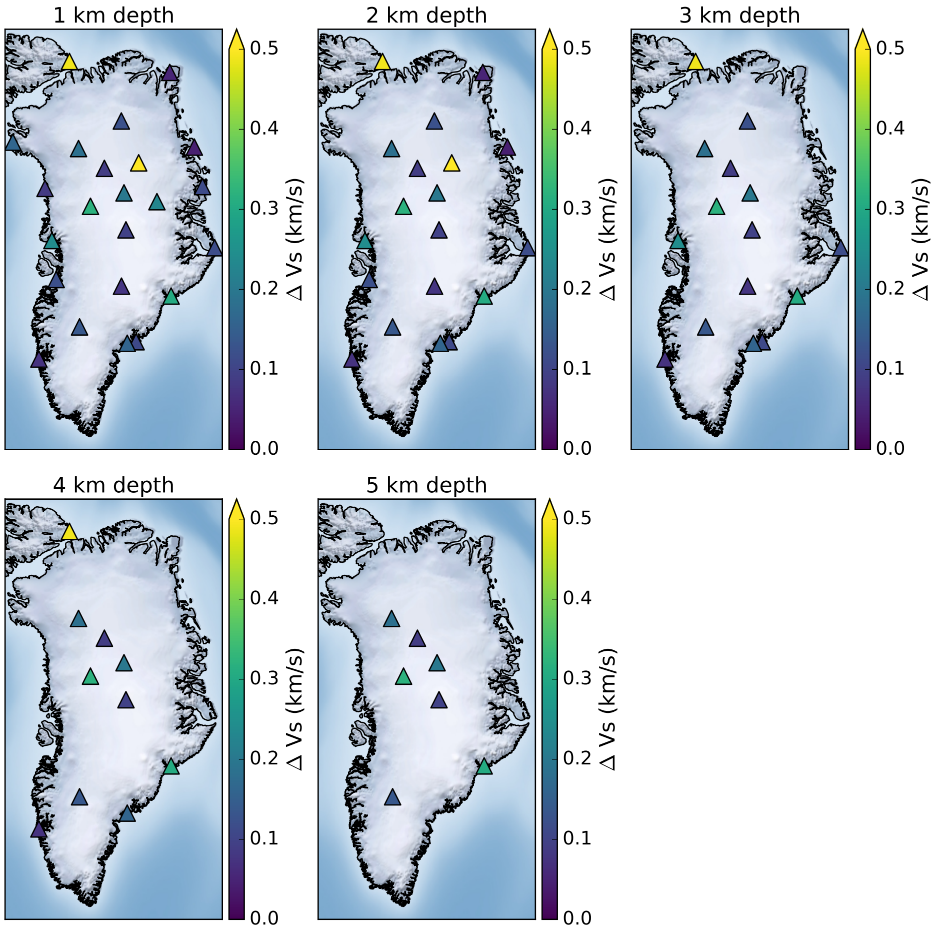

Figure 4: Uncertainty range of V_s taken from the 20% ensemble models at each depth slice.

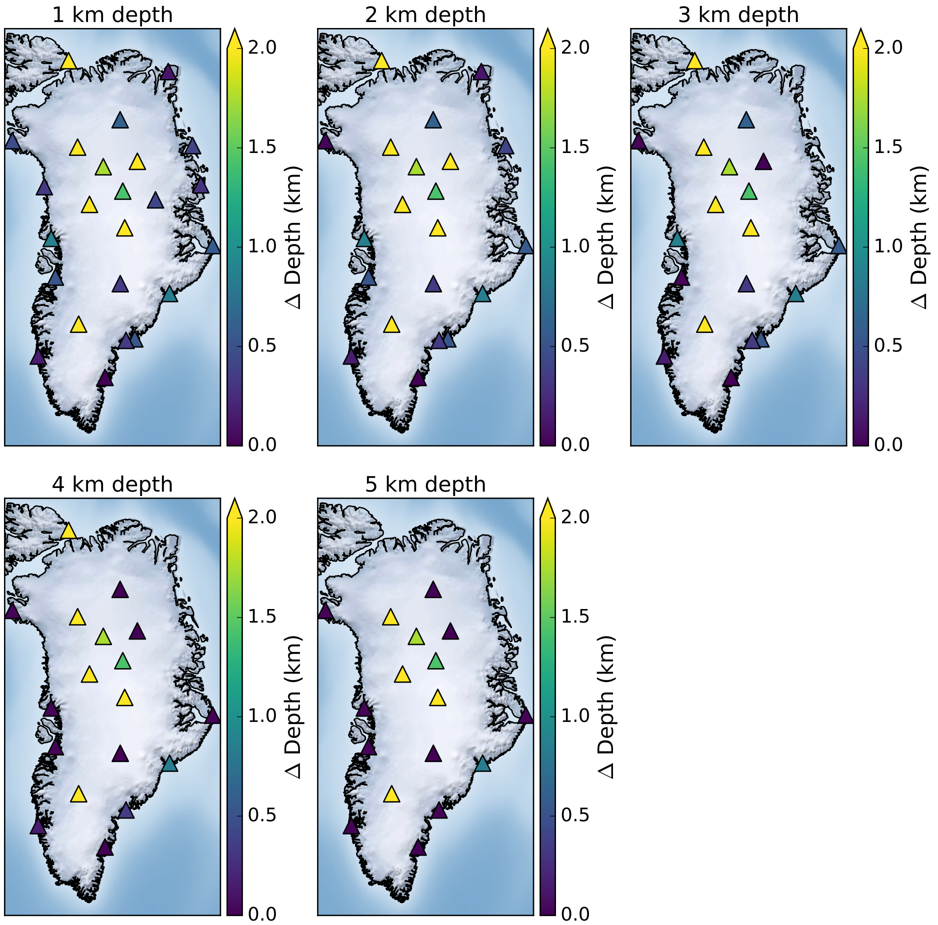

Figure 5: Depth uncertainty range taken from the 20% ensemble models at each depth slice.

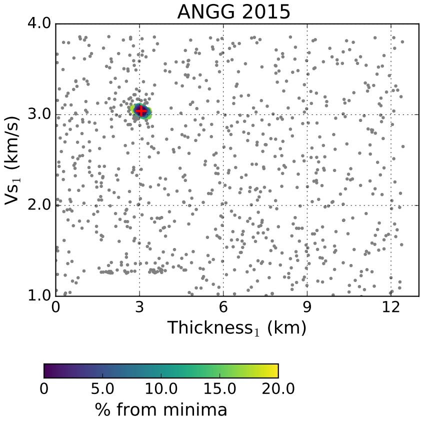

(a)

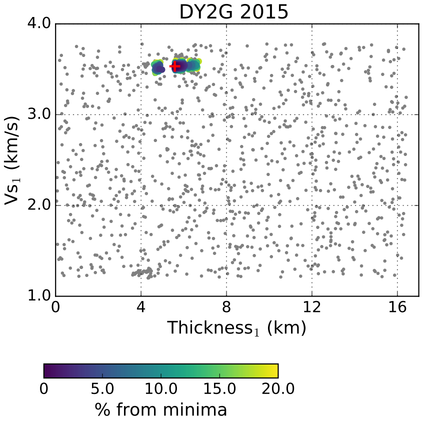

(b)

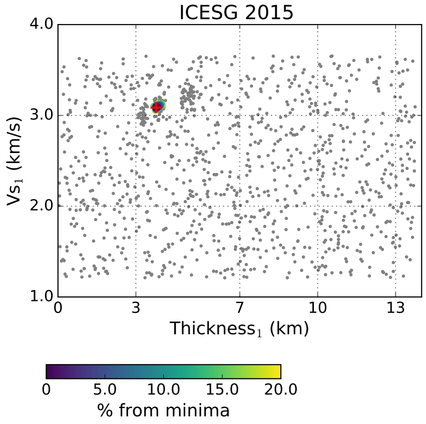

(c)

Figure 6: Scatter plot of V_s and thickness parameters for stations ANGG (no ice), DY2G (1.7 km thick ice) and ICESG (2.77 km thick ice). The best-fitting model is shown by the red cross and the parameters corresponding to solutions within 20% of this model are shown in colour. All other parameters are shown in grey. Trade-offs between parameters would appear as diagonal features.

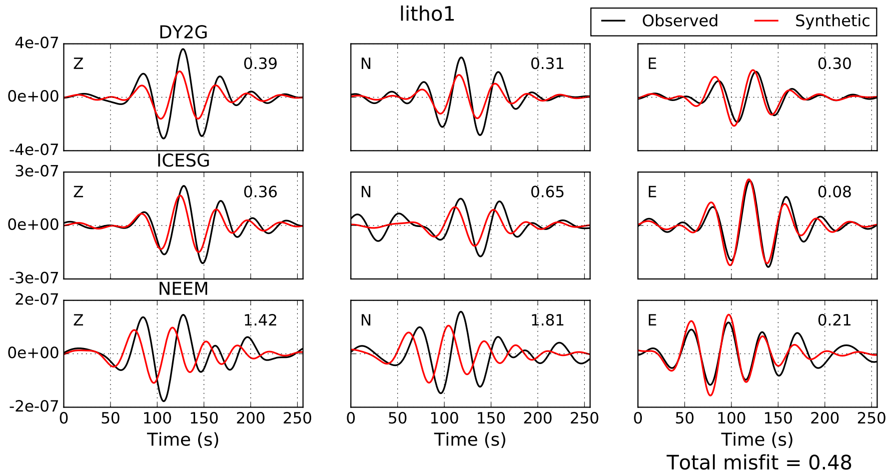

Figure 7: Comparison of theoretical (red) and observed waveform data from on-ice stations for the 11th of April 2013 Mw 4.6 south Greenland earthquake. The synthetic waveforms are calculated using CMT source parameters and the Litho1.0 model using normal mode summation implemented in the software package of (2). The numbers correspond to the L2 misfit for each individual component.

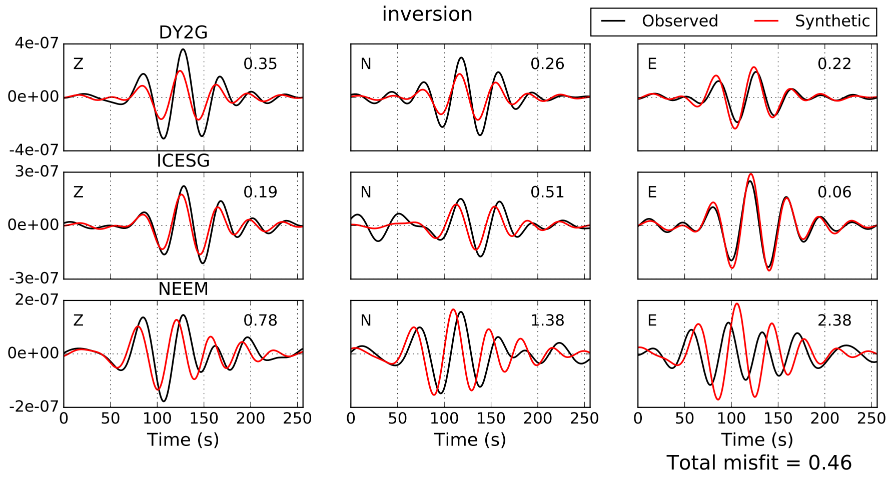

Figure 8: Comparison of theoretical (red) and observed waveform data from on-ice stations for the 11th of April 2013 Mw 4.6 south Greenland earthquake. The synthetic waveforms are calculated using CMT source parameters and our new model using normal mode summation implemented in the software package of (2). The numbers correspond to the L2 misfit for each individual component.

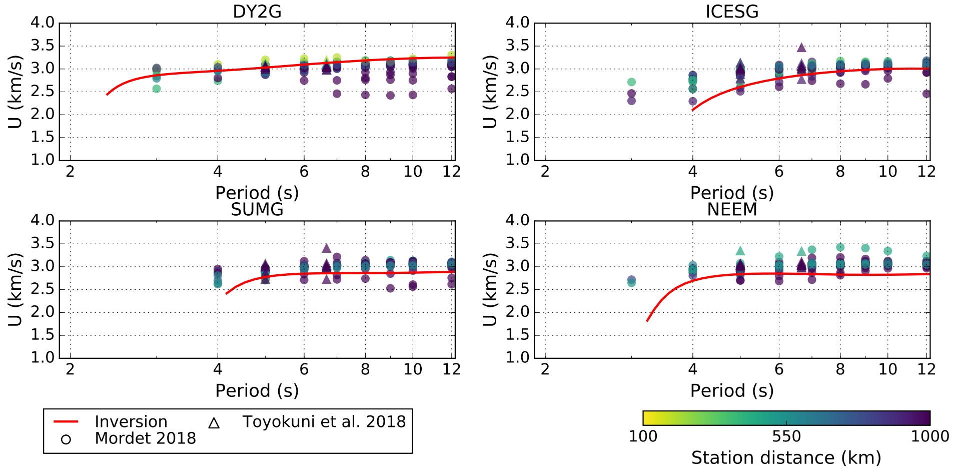

Figure 9: Comparison of group velocity estimates of the formally best fitting model from the ellipticity inversion and ambient noise measurements made by (3; 4) for on-ice stations DY2G, ICESG, SUMG and NEEM. The group velocities were computed using normal mode summation implemented in the software package of (2).

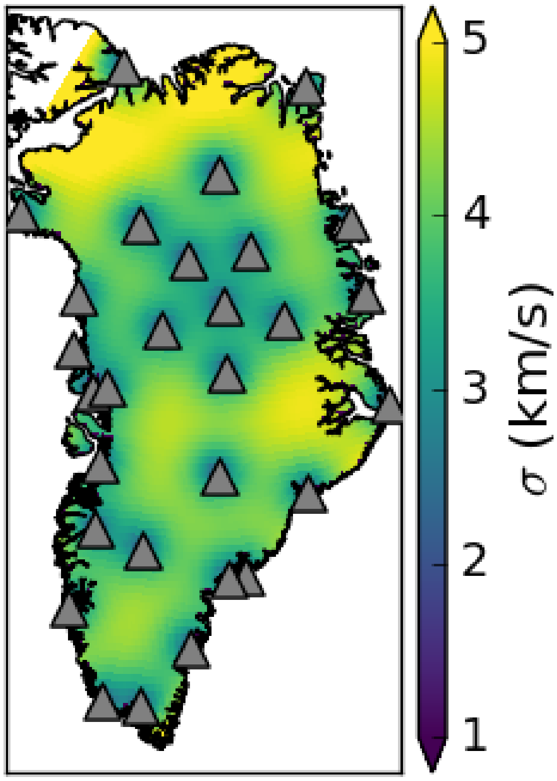

Figure 10: Standard deviation of the Kriging interpolation of V_s. Note the increase in interpolation uncertainty with increasing distance from the stations which is pronounced in the North of Greenland.

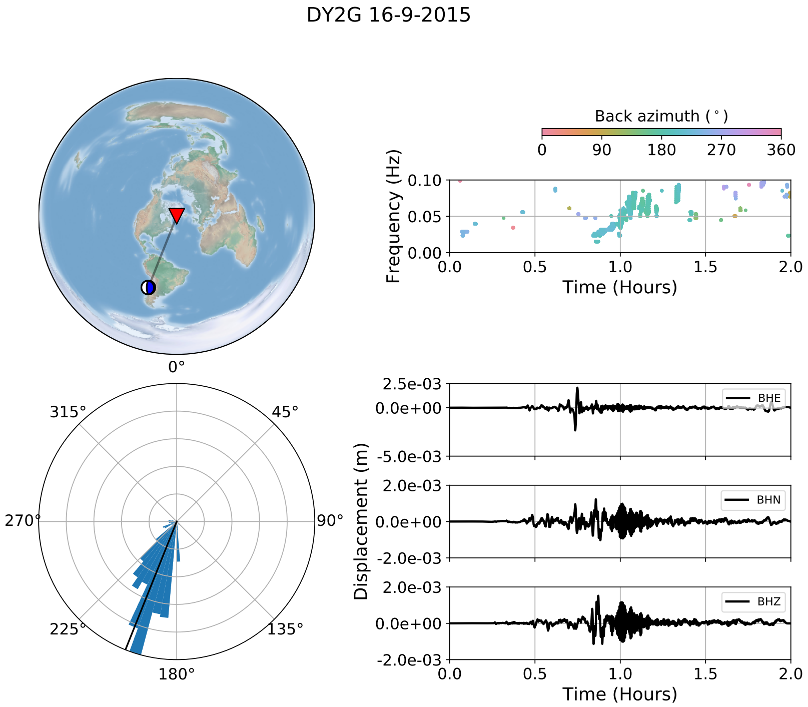

Figure 11: Example earthquake (Mw 8.3 recorded on 16 September 2015) used to calibrate the orientation of the horizontal components of the seismometer for on-ice station DY2G. Upper left: Location, source mechanism and great circle path from event to the station. The map was generated using the Python package Basemap (https://github.com/matplotlib/basemap) with public domain data (http://shadedrelief.com). Upper right: Time series of ellipticity measurements coloured by their back azimuth. Bottom left: Normalised polar histogram of the measured back azimuth with the earthquake great circle path show in black. Bottom right: Three component seismic stations. The earthuqkae was also used to determine appropriate parameters for the DOP-E method.

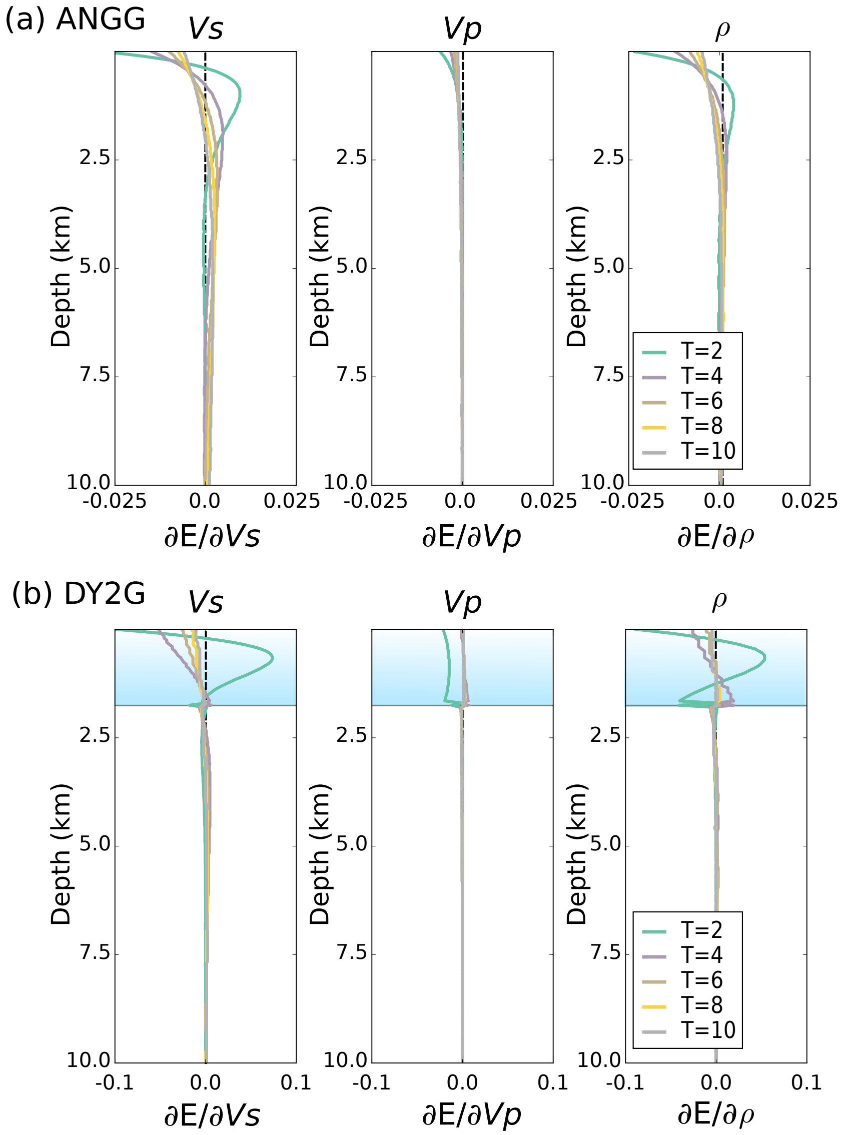

Figure 12: Ellipticity sensitivity kernels for V_s, V_p, ρ at wave periods of T=2, 4 6, 8, 10 s calculated using finite differences for the LITHO1.0 (1) for (a) an off-ice (ANGG) (b) and on-ice stations (DY2G). The top layer in (b) represents the ice.

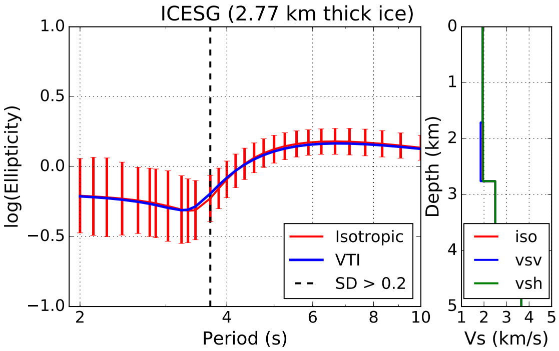

(a)

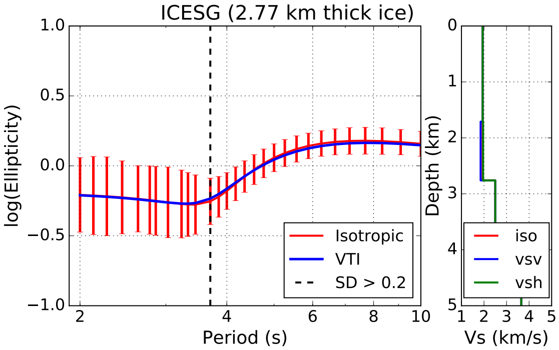

(b)

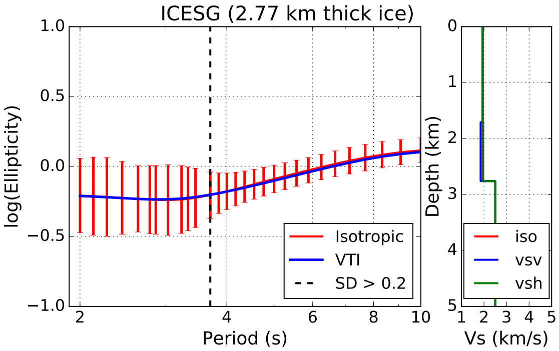

(c)

Figure 13: Comparison of modelled ellipticity curves generated using isotropic and anisotropic ice with (a) 1 km, (b) 2 km and (c) 5 km sub ice layer. Following (5) the anisotropic ice model is divided into two layers; the upper 1.71 km is parameterised as isotropic ice reflecting random orientation of ice crystals from the accumulation with the deeper ice modelled as vertical transverse isotropy corresponding to flow alignment of ice. Note the differences between both models are small and well within the measurement uncertainty shown as vertical error bars on the istotropic model.

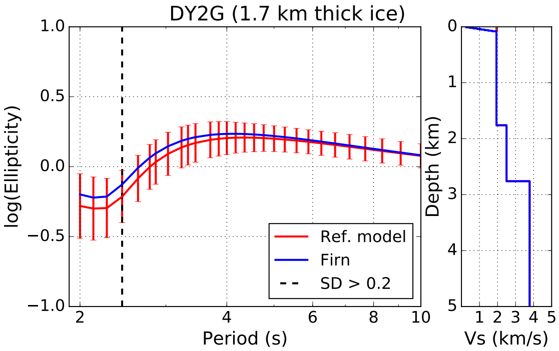

(a)

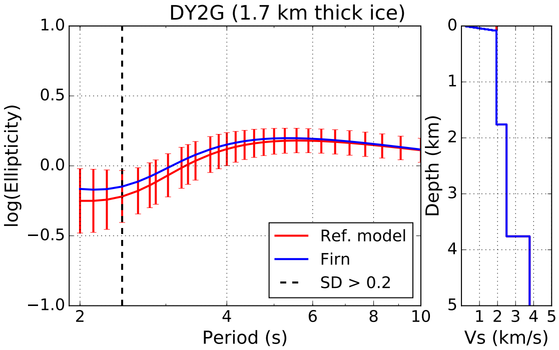

(b)

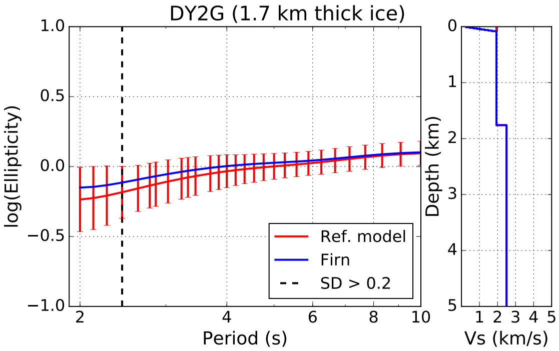

(c)

Figure 14: Comparison of modelled ellipticity curves for ice with and without firn for a (a) 1 km, (b) 2 km and (c) 5 km sub ice layer for station DY2G. We model the firn as a linear gradient (6) from the ice surface to a depth of 80 m (7) in increments of 10 m. V_p is varies from 0.5 km/s to 3.8 km/s (e.g., 8; 9) with a $V_p/V_s = 2$ (6). Density is estimated from an empirical relationship with V_p (8; 9).

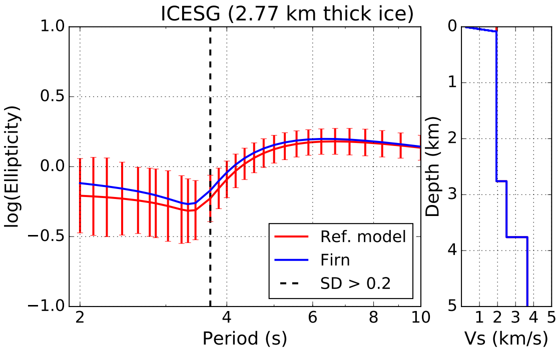

(a)

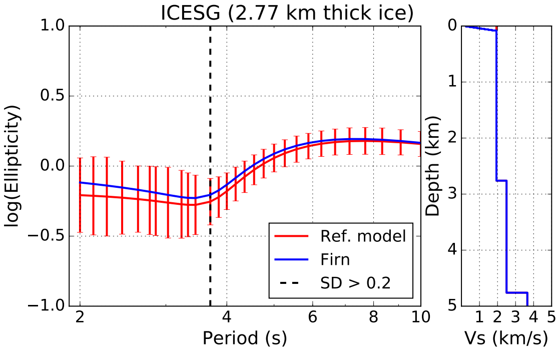

(b)

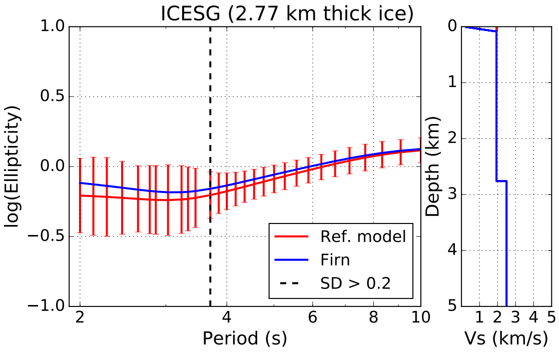

(c)

Figure 15: Comparison of modelled ellipticity curves for ice with and without firn for a (a) 1 km, (b) 2 km and (c) 5 km sub ice layer for station ICESG. We model the firn as a linear gradient (6) from the ice surface to a depth of 80 m (7) in increments of 10 m. V_p is varies from 0.5 km/s to 3.8 km/s (e.g., 8; 9) with a $V_p/V_s = 2$ (6). Density is estimated from an empirical relationship with V_p (8; 9).

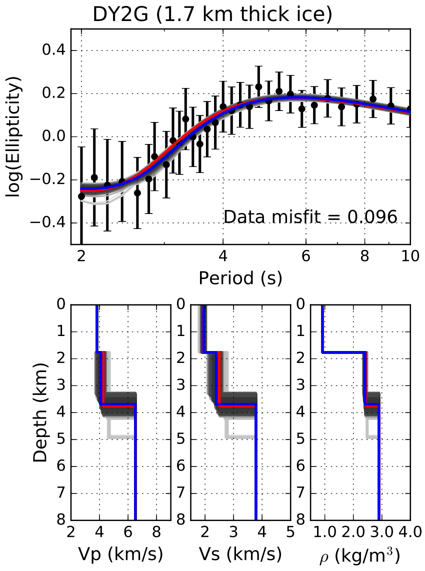

(a)

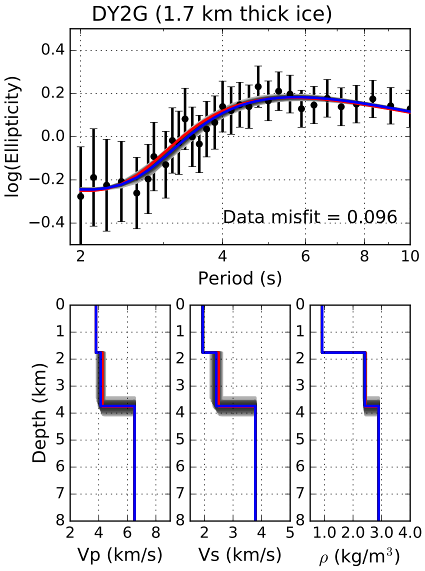

(b)

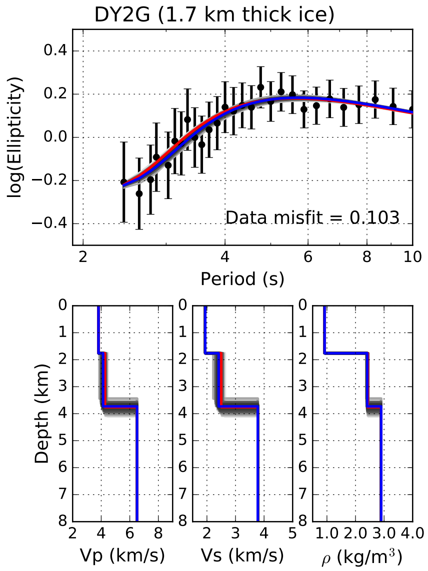

(c)

Figure 16: Results from synthetic velocity model inversion for a 2km subsurface layer for station DY2G using (a) variable V_s-ice model, (b) fixed V_s-ice model and (c) fixed V_s-ice model with ellipticity measurements with standard deviations > 0.2 removed. For each inversion the top panel compares the perturbed ellipticity data and fundamental model predictions for the true and the best fitting V_s models. The bottom three panels are 1-D depth profiles of V_p (left pane;), V_s (middle panel) and density (right panel). In all panels the blue line is the model with minimum misfit, the red line is the true model and and the grey lines correspond to the solutions from the inversions with data misfit values within 20% of the formally best-fitting model. The black dots and error bars in the top panel are the perturbed input data.

References

[1] Pasyanos, M. E., Masters, T. G., Laske, G. & Ma, Z. LITHO1. 0: An updated crust and lithospheric model of the Earth. *Journal of Geophysical Research: Solid Earth* **119**, 2153–2173 (2014).

[2] Herrmann, R. B. Computer programs in seismology: An evolving tool for instruction and research. *Seismological Research Letters* **84**, 1081–1088 (2013).

[3] Mordret, A. Uncovering the Iceland hot spot track beneath Greenland. *Journal of Geophysical Research: Solid Earth* **123**, 4922–4941 (2018).

[4] Toyokuni, G. *et al.* Changes in Greenland ice bed conditions inferred from seismology. *Physics of the Earth and Planetary Interiors* **277**, 81–98 (2018).

[5] Wittlinger, G. & Farra, V. Evidence of unfrozen liquids and seismic anisotropy at the base of the polar ice sheets. *Polar Science* **9**, 66–79 (2015).

[6] Zhan, Z., Tsai, V. C., Jackson, J. M. & Helmberger, D. Ambient noise correlation on the amery ice shelf, east antarctica. *Geophysical Journal International* **196**, 1796–1802 (2014).

[7] Riverman, K. *et al.* Enhanced firn densification in high-accumulation shear margins of the ne greenland ice stream. *Journal of Geophysical Research: Earth Surface* **124**, 365–382 (2019).

[8] Albert, D. G. Theoretical modeling of seismic noise propagation in firn at the south pole, antarctica. *Geophysical research letters* **25**, 4257–4260 (1998).

[9] Kohnen, H. On the relation between seismic velocities and density in firn and ice. *Zeitschrift für Geophysik* **38**, 925–935 (1972).
